# Genetic mapping of loci affecting seedling and adult-plant resistance to powdery mildew derived from two CIMMYT wheat lines

**DOI:** 10.1007/s00425-024-04444-9

**Published:** 2024-05-29

**Authors:** Hossein Golzar, Manisha Shankar, Beata Sznajder, Rebecca Fox, Karyn Reeves, Diane E. Mather

**Affiliations:** 1 Department of Primary Industries and Regional Development, 3 Baron Hay Ct, South Perth, WA 6151 Australia; 2 School of Agriculture and Environment, University of Western Australia, 35 Stirling Hwy, Crawley, WA 6009 Australia; 3 School of Agriculture, Food and Wine, Waite Research Institute, University of Adelaide, PMB 1, Glen Osmond, SA 5064 Australia

**Keywords:** Adult wheat plant, Pathogen resistance, Powdery mildew, Quantitative trait loci, Seedling

## Abstract

**Main conclusion:**

*PM3* and *PM8* alleles carried by two CIMMYT wheat lines confer powdery mildew resistance in seedlings and/or adult plants. A stage-specific epistatic interaction was observed between *PM3* and *PM8*.

**Abstract:**

Powdery mildew is an important foliar disease of wheat. Major genes for resistance, which have been widely used in wheat breeding programs, are typically effective against only limited numbers of virulence genes of the pathogen. The main aim of this study was to map resistance loci in wheat lines 7HRWSN58 and ZWW09-149 from the International Maize and Wheat Improvement Center (CIMMYT). Doubled haploid populations (Magenta/7HRWSN58 and Emu Rock/ZWW09-149) were developed and grown in controlled environment experiments and inoculated with a composite of *Blumeria graminis* f.sp*. tritici* isolates that had been collected at various locations in Western Australia. Plants were assessed for powdery mildew symptoms (percentage leaf area diseased) on seedlings and adult plants. Populations were subjected to genotyping-by-sequencing and assayed for known SNPs in the resistance gene *PM3*. Linkage maps were constructed, and markers were anchored to the wheat reference genome sequence. In both populations, there were asymptomatic lines that exhibited no symptoms. Among symptomatic lines, disease severity varied widely. In the Magenta/7HRWSN58 population, most of the observed variation was attributed to the *PM3* region of chromosome 1A, with the allele from 7HRWSN58 conferring resistance in seedlings and adult plants. In the Emu Rock/ZWW09-149 population, two interacting quantitative trait loci were mapped: one at *PM3* and the other on chromosome 1B. The Emu Rock/ZWW09-149 population was confirmed to segregate for a 1BL·1RS translocation that carries the *PM8* powdery mildew resistance gene from rye. Consistent with previous reports that *PM8*-derived resistance can be suppressed by *PM3* alleles, the observed interaction between the quantitative trait loci on chromosomes 1A and 1B indicated that the *PM3* allele carried by ZWW09-149 suppresses *PM8*-derived resistance from ZWW09-149, but only at the seedling stage. In adult plants, the *PM8* region conferred resistance regardless of the *PM3* genotype. The resistance sources and molecular markers that were investigated here could be useful in wheat breeding.

**Supplementary Information:**

The online version contains supplementary material available at 10.1007/s00425-024-04444-9.

## Introduction

Powdery mildew caused by *Blumeria graminis* f.sp. *tritici* (syn. *Erysiphe graminis* f. sp. *tritici*) is an important foliar disease of common wheat (*Triticum aestivum* L.) worldwide. Yield loss, which depends on the level of resistance in wheat varieties and on environmental factors, has been reported to range from 20 to 33% in United States (Fried et al. 1981; Kingsland 1982; Leath and Bowen 1989; Griffey et al. 1993). In Western Australia, powdery mildew occurs sporadically on susceptible wheat cultivars, causing economic yield losses. Although the disease is of economic significance mainly in high-rainfall areas, its severity in hotter, drier areas has increased due to use of susceptible varieties and increased rates of nitrogen fertiliser.

Breeding for resistance to powdery mildew is an effective and environmentally safe approach in preventing yield losses, especially if multiple disease resistance genes are deployed to combat changes in pathogen virulence. Major genes, which have been widely used in wheat breeding programs, are typically effective against only limited numbers of *B. graminis* f.sp. *tritici* virulence genes. Widespread deployment of varieties with this type of resistance imposes strong selection pressure on the pathogen population, in favour of the corresponding virulence genes (Szunics et al. 1999).

To date, about 90 genes for powdery mildew resistance, at 60 wheat loci, have been reported (McIntosh et al. 2001, 2013, 2018; Liu et al. 2002; Miranda et al. 2006; Hao et al. 2014; Zhang et al. 2019). For some resistance loci, including *PM3* and its rye-derived orthologues *PM8* and *PM17*, the causal genes have been isolated and found to encode nucleotide-binding-site leucine-rich repeat (NLR) proteins (Yahiaoui et al. 2004; Hurni et al. 2013; Singh et al. 2018). Many alleles of *PM3* have been isolated (Yahiaoui et al. 2006; Bhullar et al. 2010). Despite high sequence conservation, these alleles confer highly specific resistance because the NLRs they encode can differentiate among structurally similar effectors (Bourras et al. 2019). Among these alleles, *Pm3a* and *Pm3e* have been postulated to be effective in Western Australia (Golzar et al. 2016).

Although most reported resistance genes are race specific, some confer non-specific partial resistance (Lillemo et al. 2008; Zhang et al. 2018). Partial resistance has been reported to extend latent period, slowing disease expression, reducing sporulation, and consequently reducing yield loss (Bennett 1984; Hautea et al. 1987). Due to the difficulty of selecting for partial resistance (Shaner and Finney 1975; Gustafson and Shaner 1982), marker-based selection for this type of resistance could be useful (Lan et al. 2010; Ma et al. 2011; Bai et al. 2012; Asad et al. 2013). This requires identification of marker-trait associations (MTAs). For powdery mildew resistance, MTAs have been mapped on various wheat chromosomes using quantitative trait locus (QTL) analysis in bi-parental mapping populations (Liu et al. 2017; Li et al. 2019) and genome-wide association studies (GWAS) in diversity panels (Nelson et al. 2018; Kang et al. 2020).

The objective of the research reported here was to genetically map loci that confer powdery mildew resistance in two wheat lines from the International Maize and Wheat Improvement Center (CIMMYT).

## Materials and methods

### Plant materials and fungal isolates

The resistance sources used in this research were 7HRWSN58 (CIMMYT 7th High Rainfall Wheat Screening Nursery entry 58, pedigree ASIO/3/F6.74/BUN//SIS) and ZWW09-149 (synonyms ZWW09-149, ZWW09Q149 and 18SAWYT149 (CIMMYT 18th Semi-Arid Wheat Yield Trial entry 149); pedigree PBW343*2/KHVAKI//PASTOR/SLVS). Each of these lines was used as the paternal parent in a cross with an Australian wheat variety: 7HRWSN58 with Magenta (pedigree Carnamah/Tammin-18) and ZWW09-149 with Emu Rock (pedigree 96W657-37/Kukri). Magenta is moderately resistant to moderately susceptible to powdery mildew in Western Australia, and Emu Rock is moderately susceptible to susceptible. Different varieties were used as parents to have more diversity in the two doubled haploid (DH) populations which is advantageous for breeding programs aiming to broaden the genetic base of cultivated wheat varieties. For each cross, a set of F_1_-derived DH lines was generated using a wheat/maize pollination system (Broughton et al. 2014).

Forty *B. graminis* f.sp. *tritici* isolates, collected from various locations in Western Australia over five years, were composited and propagated on the susceptible cultivar Federation in a controlled environment at 18 ± 2 °C with a 12-h dark and light cycle until 50% of the leaf surface was colonised. Inoculations on test lines were performed by dusting conidia propagated from the composite of 40 isolates on susceptible seedlings of Federation (Bapela et al. 2023).

### Phenotyping

The Magenta/7HRWSN58 population was phenotyped in 2016 and 2017. The Emu Rock/ZWW09-149 population was phenotyped in 2018 and 2019. All trials were conducted in a growth room at 18 ± 2 °C with a 12-h dark and light cycle using randomised complete block designs with two blocks. For each trial, four seeds of each line were sown into each of two 150-mm-diameter pots (one per block) containing commercial potting mix (two parts river sand and one-part peat moss with nutrients and trace elements). At Zadoks growth stage 13 (seedlings with three leaves emerged; Zadoks et al. 1974) and at 2–3 week intervals thereafter, inoculum was evenly dusted on each plant. At 10 days after the first inoculation, percentage leaf area diseased was estimated for each seedling. At Zadoks growth stage 55 (first spike half-emerged from the boot), percentage leaf area diseased was estimated on the flag and flag-1 leaves of the main stem of each plant.

### Genotyping and linkage mapping

Leaf tissue was sampled from individual seedlings of each DH line and parent. Genomic DNA was isolated using a phenol chloroform method (Rogowsky et al. 1991) with modifications as described by Pallotta et al. (2000). The resulting DNA samples were analysed by Diversity Arrays Technology (Bruce, ACT, Australia) using its DArTseq genotyping-by-sequencing platform (www.diversityarrays.com/dart-application-dartseq) and analysis software. Single-nucleotide polymorphisms (SNPs) and SilicoDArT presence–absence polymorphisms were scored. Where possible, sequence tags were anchored to physical positions on pseudomolecules in Version 2.1 of the Chinese Spring wheat reference genome sequence assembly (Zhu et al. 2021) using BLAST (Altschul et al. 1990; e-value 1e-10, maximum target sequences 1). Seven competitive allele-specific PCR (KASP) assays (Table S1) were designed for SNPs among known alleles of the *PM3* gene (Yahiaoui et al. 2004; Srichumpa et al. 2005; Tomasini et al. 2006) and applied to Magenta, 7HRWSN58, Emu Rock and ZWW09-149, using an automated SNPLine system (LGC Limited, Teddington, UK). Three of these assays (for the SNPs Pm3CS_C1651G, Pm3CS_G1693A and Pm3CS_T3155A) were applied to the Magenta/7HRWSN58 and Emu Rock/ZWW09-149 populations. In addition, to test for a 1BL·1RS translocation chromosome that is known to be present in an ancestor of ZWW09-149 (PBW343; personal communication, Richard Trethowan, University of Sydney), presence-absence assays for the wheat *GLI-B1* and *GLU-B3* genes and for rye-derived $\omega$-secalin, pAWRC.1 and *PM8* sequences were applied to Emu Rock, ZWW09-149 and seven Emu Rock/ZWW09-149 lines using the primers listed in Table S2 and the methods described by Ren et al. (2017) (*GLI-B1*), Chai et al. (2006) (*GLU-B3* and $\omega$-secalin), Yang et al. (2014) (pAWRC.1) and Hurni et al. (2013) (*PM8*). The *PM8* assay was also applied to the Emu Rock/ZWW09-149 population.

Genotypic data were analysed in the R Statistical Computing Environment (R Core Team 2023), using the packages R/qtl (Broman et al. 2003) and R/ASMap (Taylor and Butler 2016). Individual markers were excluded from the data set if their minor allele frequency was less than 0.05, or if more than 20% of their genotypic values were missing. Similarly, individual DH lines were excluded if more than 20% of their genotypic values were missing. The remaining genotypic data were investigated to identify pairs of lines with identical or nearly identical results (sharing the same alleles for more than 95% of the markers for which data were available for all lines). For each pair of lines discovered in this way, the individual line genotypes were collapsed to a consensus genotype (Taylor and Butler 2016) and the individual line names were concatenated to provide a consensus line name. Linkage maps were constructed using the MSTmap algorithm (Wu et al. 2008) as implemented in R/ASMap. Linkage groups were assigned to chromosomes and oriented based on estimated physical positions of sequence tags in the reference genome assembly.

### Statistical analysis

For each line, means were calculated for the percentage leaf area diseased for seedlings and for adult plants, across plants within experimental units and blocks within trials (years). Spearman rank correlation coefficients (*r*_s_) were calculated between the years. Overall means (across years) were calculated and used as the phenotypic values for QTL analysis. QTL analysis was conducted by simple interval mapping, with imputation of missing genotypic data as implemented in R/qtl. Significance of LOD test statistic values was evaluated relative to thresholds obtained using 10,000 permutations and a genome-wide significance level of 0.05.

## Results

### Magenta/7HRWSN58

**Fig. 1:**
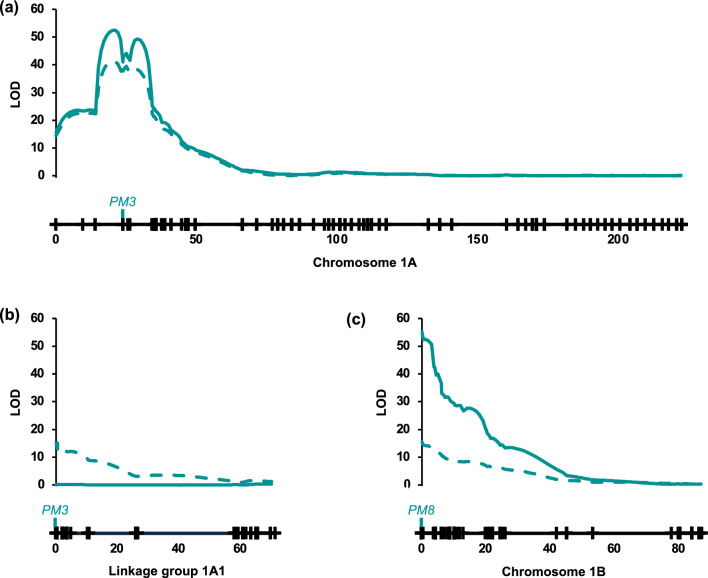
Linkage maps (including the positions of the powdery mildew resistance loci *PM3* and *PM8*) and LOD test statistic values for chromosome 1A (**a**), as mapped using a Magenta/7HRWSN58 wheat population, the main linkage group of chromosome 1A (**b**), as mapped using an Emu Rock/ZWW09-149 wheat population, and chromosome 1B (**c**), as mapped using an Emu Rock/ZWW09-149 wheat population. Numbers on linkage maps indicate positions in cM. LOD scans for % leaf area diseased for seedlings and adult plants are shown with dashed and solid lines, respectively. Complete linkage map information including marker names and positions is shown in Tables S3 and S4

**Fig. 2:**
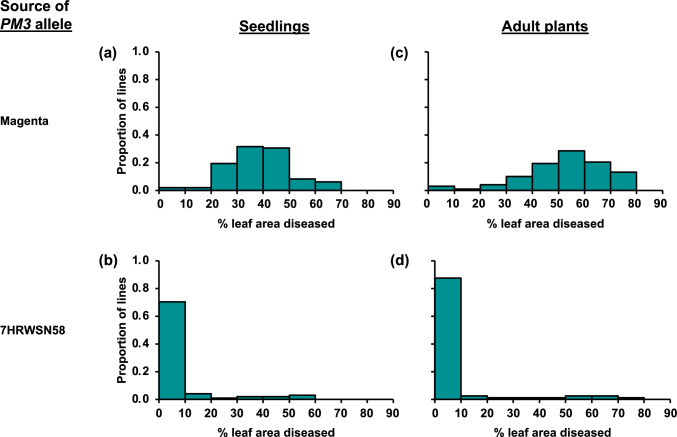
Phenotypic distributions for mean % leaf area diseased on seedlings (**a** and **b**) and adult plants (**c** and **d**) for Magenta/7HRWSN58 wheat lines classified according to whether they carry Magenta alleles or 7HRWSN58 alleles at the *PM3* locus

In the Magenta/7HRWSN58 population, the distributions of phenotypic values deviated strongly from normality, with many lines exhibiting no disease symptoms (Table S3). Rankings of lines according to disease severity were quite consistent across trials (*r*_*s*_ = 0.87 (*P* < 0.0001) for seedlings; *r*_*s*_ = 0.90 (*P* < 0.0001) for adult plants). Among the lines for which symptoms were observed, mean phenotypic values (calculated across blocks and trials) ranged from just above 0% to maxima of 65.0% for seedlings and 75.0% for adult plants. Across *PM3* SNPs that were genotyped with KASP assays, the haplotypes of Magenta and 7HRWSN58 differed from each other and from those of known *PM3* alleles (Table S1). The linkage map constructed for the Magenta/7HRWSN58 population consisted of 2802 markers (2799 DArTseq SNPs and three *PM3* SNPs) on 25 linkage groups (two for each of chromosomes 5D, 6B, 6D and 7D and one for each of the other 17 chromosomes in the wheat genome) (Table S3). The three *PM3* SNPs co-segregated with each other, mapping at 23.8 cM on chromosome 1A. Just distal to this position, a very highly significant QTL was mapped in the Magenta/7HRWSN58 population (Fig. 1, Table 1). Almost all lines with the Magenta *PM3* allele exhibited symptoms as seedlings (Fig. 2a) and as adult plants (Fig. 2c). Almost all lines carrying the 7HRWSN58 *PM3* allele exhibited few or no symptoms (Fig. 2b and d).

**Table 1:** Quantitative trait loci (QTLs) for powdery mildew resistance mapped based on % leaf area diseased in seedlings and adult plants in Magenta/7HRWSN58 and Emu Rock/ZWW09-149 wheat populations

Population	Chromosome (and linkage group)	QTL position (cM)	Growth stage	LOD score	Source of resistance allele	Closest markers
Magenta/7HRWSN58	1A	20.0	Seedling	41.4*	7HRWSN58	Pm3CS_C1651G, Pm3CS_G1693A and Pm3CS_T3225A
		21.0	Adult	52.5*	7HRWSN58	Pm3CS_C1651G, Pm3CS_G1693A and Pm3CS_T3225A
Emu Rock/ZWW09-149	1A (1A1)	0.5	Seedling	15.1*	Emu Rock	Pm3CS_C1651G, Pm3CS_G1693A, Pm3CS_T3225A and 24 DArTseq SNPs
	1B	0.0	Seedling	15.5*	ZWW09-149	*PM8* and 144 DArTseq SNPs
			Adult	55.2*	ZWW09-149	*PM8* and 144 DArTseq SNPs

*Significant at a genome-wide significance level of 0.05

### Emu rock/ZWW09-149

In the Emu Rock/ZWW09-149 population, the distributions of phenotypic values deviated strongly from normality, with many lines exhibiting no disease symptoms (Table S4). Rankings of lines according to disease severity were quite consistent across the two trials (*r*_*s*_ = 0.72 (*P* < 0.0001) for seedlings; *r*_*s*_ = 0.88 (*P* < 0.0001) for adult plants). Among the lines for which symptoms were observed, mean phenotypic values (calculated across blocks and trials) ranged from just above 0% to maxima of 67.0% for seedlings and 59.8% for adult plants.

Across *PM3* SNPs that were genotyped with KASP assays, the haplotypes of Emu Rock and ZWW09-149 differed from each other, from those of Magenta and 7HRWSN58, and from those of 35 known *PM3* alleles (Table S1). With 1BS-specific *Gli-B1* and *Glu-B3* marker assays, amplicons were obtained for Emu Rock but not for ZWW09-149 (Fig. S1a, b). With 1RS-specific $\omega$-secalin, pAWRC.1 and *PM8* marker assays, amplicons were obtained for ZWW09-149 but not for Emu Rock (Fig. S1b–d).

The linkage map constructed for the Emu Rock/ZWW09-149 population consisted of 4,596 markers (three *PM3* SNPs, one *PM8* marker and 4592 DArTseq SNPs) on 25 linkage groups (two for each of chromosomes 1A, 2D, 3B, 3D and 4D, and one for each of the other 16 chromosomes in the wheat genome) (Table S4). The three *PM3* SNPs collocated with each other and with 24 DArTseq SNPs at 0.5 cM on linkage group 1A1. Of the 24 DArTseq reference sequence tags with SNPs mapping at that position, 17 (85.0%) were anchored to the 1A pseudomolecule of the reference genome assembly, at positions ranging from 1.9 to 13.3 Mb, with seven distal to *PM3* (5.5 Mb) and ten proximal to *PM3* (Table S4). The *PM8* presence-absence marker cosegregated with 140 DArTseq SNPs at 0.0 cM on chromosome 1B. Of the 144 DArTseq reference sequence tags with SNPs mapping at that position, 78 (54.2%) were anchored to the 1B pseudomolecule of the wheat reference genome assembly, at positions ranging from 3.7 to 229.3 Mb, 29 (20.1%) were anchored to other wheat pseudomolecules and 37 (25.7%) were not anchored to the wheat reference genome assembly (Table S4). There were also 1,157 SilicoDArT sequence tags (presence-absence markers) that cosegregated with *PM8*: 645 Emu Rock-specific sequences (Table S5) and 512 ZWW09-149-specific sequences (Table S6). Among the Emu Rock-specific sequences, 408 (63.3%) were anchored to the 1B pseudomolecule and 196 (30.4%) could not be anchored on any pseudomolecule. In contrast, among the ZWW09-149-specific sequences, only 26 (5.1%) were anchored to the 1B pseudomolecule, while 353 (68.9%) could not be anchored to any pseudomolecule.

Two QTLs were mapped in the Emu Rock/ZWW09-149 population, one at the *PM3* position on chromosome 1A and the other at the *PM8* position on chromosome 1B (Fig. 1b, Table 1). The QTL on chromosome 1A was detected only in seedlings, while the QTL on chromosome 1B was detected at both stages. The *PM8* region exhibited strong segregation distortion (${\text{X}}^{2}=16.5$; $P<0.0001$) in favour of alleles from ZWW09-149.

**Fig. 3:**
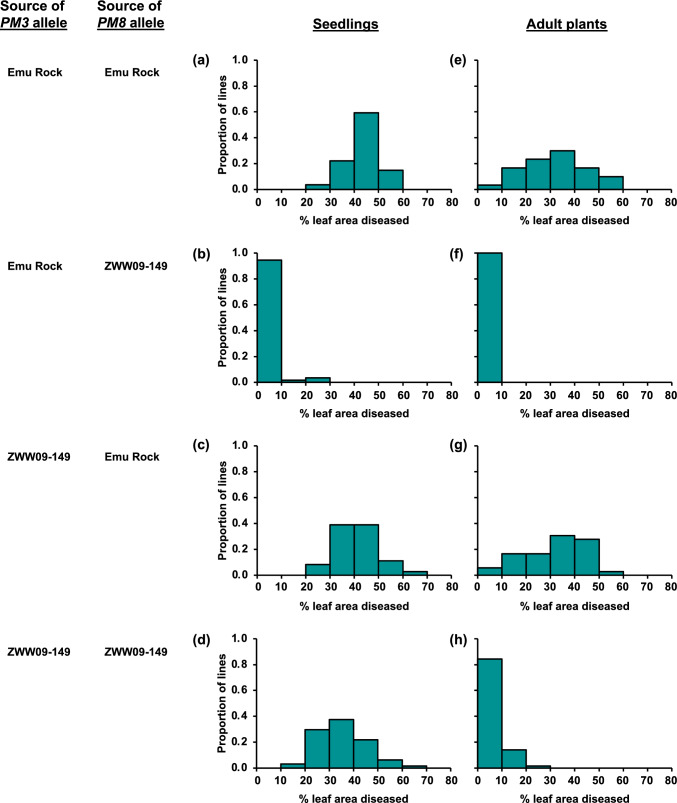
Phenotypic distributions for mean % leaf area diseased on seedlings (**a**–**d**) and adult plants (**e**–**h**) for Emu Rock/ZWW09-149 wheat lines classified into four groups based on whether they carry Emu Rock or ZWW09-149 alleles at the *PM3* and *PM8* loci

Examination of the distributions of phenotypic values within each of the four genotypic classes defined by marker genotypes at the two QTL positions revealed a strong interaction between the QTLs at the seedling stage (Fig. 3a–d), with resistance observed only for lines with Emu Rock alleles at *PM3* and ZWW09-149 alleles at *PM8* (Fig. 2b). No such interaction was observed at the adult stage (Fig. 3e–h), at which resistance was observed for both genotypic classes with the ZWW09-149 genotype at *PM8* (Fig. 3f and h).

## Discussion

The results of this genetic mapping show that that the powdery mildew resistance of CIMMYT wheat line 7HRWSN58 is due to a locus on chromosome 1A, while that of CIMMYT wheat line ZWW09-149 is affected by loci on chromosomes 1A and 1B. In both mapping populations, the position of the locus on chromosome 1A coincides with that of the multi-allelic powdery mildew locus *PM3*. In the Emu Rock/ZWW09-149 population, the position of the locus on chromosome 1B coincides with that of *PM8*, which is the rye orthologue of *PM3* (Hurni et al. 2013). This result, in combination with the co-segregation of *PM8* with many DArTseq SNPs and presence-absence markers indicates that ZWW09-149 carries a 1BL·1RS translocation chromosome, which it would have inherited from PBW343. This translocation is also known to confer agronomic advantages (Zhao et al. 2012; Moskal et al. 2021). These may explain the segregation distortion that was observed here, which favoured the 1RS chromosome arm over the 1BS chromosome arm. Unfortunately, the 1RS chromosome arm also has negative effects on wheat end-use quality (Zhao et al. 2012; Moskal et al. 2021), which limit the utility of 1BL·1RS translocation lines for baking or noodle production.

In our experiments, the 7HRWSN58 resistance was effective at both seedling and adult stages, while the resistance of ZWW09-149 was affected by a stage-specific epistatic interaction. The seedling stage interaction is consistent with reports by McIntosh et al. (2011), Hao et al. (2012) and Hurni et al. (2014) that *PM3* alleles can suppress *PM8*-mediated resistance to powdery mildew, with the occurrence and strength of the suppression varying among *PM3* alleles and among isolates of the pathogen. These reports were based on evaluation of resistance on seedlings and/or detached leaves.

In the Emu Rock/ZWW09-149 population, seedling resistance was observed only in the genotypic class that carries the ZWW09-149 allele at *PM8* and the Emu Rock allele at *PM3* (Fig. 2b), indicating that the ZWW09-149 *PM3* allele acts as a suppressor, but the Emu Rock *PM3* allele does not. This is consistent with previous reports of suppression of *PM8* resistance by *PM3* alleles (McIntosh et al. 2011; Hurni et al. 2014). According to Hurni et al. (2014), this suppression involves post-translational interaction of PM3 and PM8 proteins. Similarly, Stirnweis et al. (2014) reported post-translational suppression among the products of *PM3* alleles. These previous reports are all based on interactions observed in seedlings and/or at the cellular level. In the research reported here, suppression occurred in seedlings, but not in adult plants. Based on this observation, it could be useful to investigate the developmental specificity of PM3*-*PM8 protein–protein interaction. Even without understanding of the mechanism, this observation could be useful in wheat breeding, as it indicates that adult-stage resistance is possible even for allelic combinations that do not confer resistance in seedlings.

### Supplementary Information

Below is the link to the electronic supplementary material.

Supplementary file1 (XLSX 5574 KB)

Supplementary file2 (PDF 1673 KB)

## Data Availability

The genotypic and phenotypic data used for genetic analysis are provided in Tables S3 and S4.

## Abbreviations

DH: Doubled haploid
QTL: Quantitative trait locus
SNP: Single-nucleotide polymorphism
