# Supplementary material for: Genetic mapping of loci affecting seedling and adult-plant resistance to powdery mildew derived from two CIMMYT wheat lines
Source: Planta. 2024 May 29;260(1):13. doi: 10.1007/s00425-024-04444-9 (PMC11136728; doi:10.1007/s00425-024-04444-9)
Supplement: Supplementary file 2 — Supplementary file2 (PDF 1673 KB) [file 425_2024_4444_MOESM2_ESM.pdf]

Online Resource 2: Supplementary Figure 1

**Article title:**	Genetic mapping of loci affecting seedling and adult plant resistance to powdery mildew derived from two CIMMYT wheat lines
**Journal:**	Planta
**Authors:**	Hossein Golzar, Manisha Shankar, Beata Sznajder, Rebecca Fox, Karyn Reeves and Diane Mather
**Corresponding author:**	Manisha Shankar, School of Agriculture and Environment, University of Western Australia, 35 Stirling Hwy, Crawley, Western Australia 6009, Australia; Department of Primary Industries and Regional Development, 3 Baron Hay Ct, South Perth, Western Australia 6151, Australia; manisha.shankar@uwa.edu.au; manisha.shankar@dpird.wa.gov.au

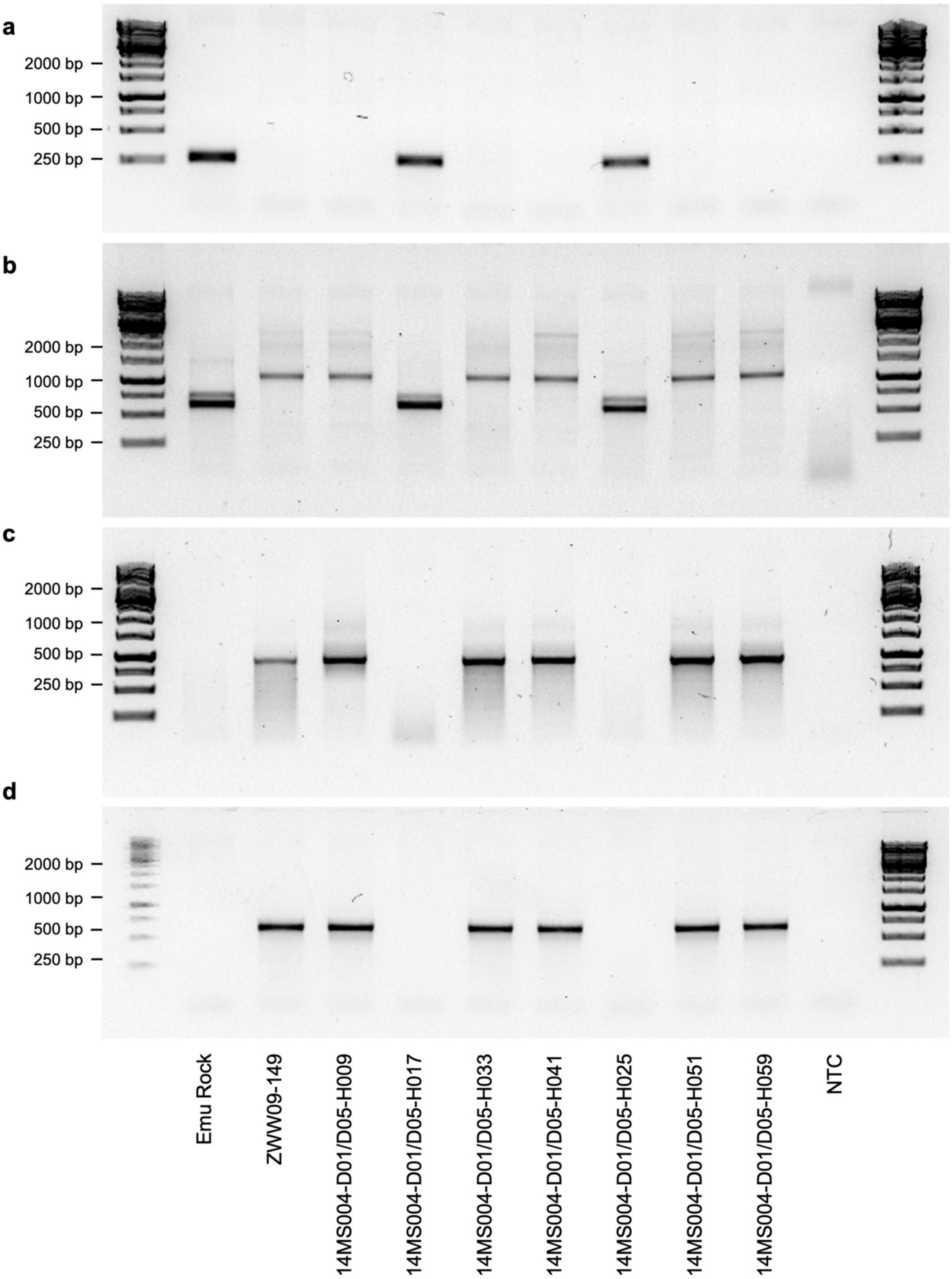

Fig. S1 Agarose gel electrophoresis (1% agarose) of products amplified from Emu Rock, ZWW09-149, seven Emu Rock/ZWW09-149 doubled haploid lines and a no-template control (NTC) using primer sets specific to (**a**) wheat *GLI-B1*, (**b**) wheat *GLU-B3* (630 bp product) and rye ω-secalin (1100 bp product), (**c**) rye pAWRC.1 and (**d**) rye *PM8*
